# Active Surface-Enhanced Raman Scattering Platform Based on a 2D Material–Flexible Nanotip Array

**DOI:** 10.3390/bios14120619

**Published:** 2024-12-15

**Authors:** Yong Bin Kim, Satyabrat Behera, Dukhyung Lee, Seon Namgung, Kyoung-Duck Park, Dai-Sik Kim, Bamadev Das

**Affiliations:** 1 Department of Physics, Pohang University of Science and Technology (POSTECH), Pohang 37673, Republic of Korea; life0944@postech.ac.kr (Y.B.K.); parklab@postech.ac.kr (K.-D.P.); 2 Department of Physics and Quantum Photonics Institute, Ulsan National Institute of Science and Technology (UNIST), Ulsan 44919, Republic of Korea; satyabrat2020@unist.ac.kr (S.B.); dukhyung.lee@um6p.ma (D.L.); seon@unist.ac.kr (S.N.)

**Keywords:** tunable SERS, multi-order Raman scattering, MoS_2_ flexible nano-tip, enhancement factor

## Abstract

Two-dimensional materials with a nanostructure have been introduced as promising candidates for SERS platforms for sensing application. However, the dynamic control and tuning of SERS remains a long-standing problem. Here, we demonstrated active tuning of the enhancement factor of the first- and second-order Raman mode of monolayer (1L) MoS_2_ transferred onto a flexible metallic nanotip array. Using mechanical strain, the enhancement factor of 1L MoS_2_/nanotip is modulated from 1.23 to 8.72 for 2LA mode. For the same mode, the SERS intensity is enhanced by ~31 times when silver nanoparticles of ~13 nm diameter are deposited on 1L MoS_2_/nanotip, which is tuned up to ~34 times by compressive strain. The change in SERS enhancement factor is due to the decrease (increase) in gap width as the sample is bent inwardly (outwardly). This is corroborated by FEM structural and electromagnetic simulation. We also observed significant control over mode peak and linewidth, which may have applications in biosensing, chemical detection, and optoelectronics.

## 1. Introduction

Surface-enhanced Raman scattering (SERS) is an enhanced version of Raman scattering enabled by metallic nanostructure. It is now well documented that an enhanced Raman signal, i.e., SERS can be attributed to electromagnetic and chemical enhancement. While the chemical enhancement mainly comes from the interaction between the molecule and the metallic substrate, the architecture of the substrates plays an important role towards electromagnetic enhancement [1,2]. Metallic nanostructure-decorated substrates [3] such as nanospheres or nanoparticles [4,5], nanorods [5,6], nanowires [6], nano-stars [7,8], and nanoscale-roughened films [9,10] have enabled application in biosensing [11,12], pesticide detection [13], and chemical analysis [14]. Apart from the metallic nanostructure, plasmonic structures such as nanogap have also shown great SERS enhancement [15,16,17]. However, research into a dynamic SERS platform pivoted by tunable electromagnetic field localization is still lagging. Recently, enormous amounts of research have been focused to develop a highly sensitive SERS platform based on two-dimensional (2D) materials [15,18,19]. Among 2D materials, transition metal dichalcogenides (TMDs) are an emerging class of materials with superior applications in opto- electronics. In particular, their dynamic response to external perturbations such as mechanical strain [20], electrostatic gating [21], and pressure [22] makes them a great candidate for a flexible SERS platform [8,9]. Amongst various external perturbation methods, mechanical strain is an easy and exciting way to control the electronics properties, band gap, and phonon dispersion of TMDs, facilitating applications in tunable photonics devices [23], flexible electronics [21], thermoelectric energy conversion [24], field effect transistor [25], and catalysis [26]. MoS_2_–metal hybrid structures have been reported to show improved optical properties [27]. When 1L MoS_2_ is decorated on the nanostructure by means of mechanical exfoliation or chemical vapor deposition (CVD), intrinsic strain is generated on the monolayer, which dynamically changes the band-gap and its phonon properties.

This kind of system can have a dynamic response due to laser pressure [25], which lacks reconfigurable functionality and controllability. Moreover, most of these studies have particularly focused on opto-electronics device application. Hence, an active system that can dynamically control the SERS intensity of 2D materials needs to be designed. Here, we report a system consisting of mechanically exfoliated 1L MoS_2_ on a flexible nanotip array. We could dynamically control the electromagnetic field that is confined in between the two neighboring nanotips by applying external mechanical strain, which would then tune the SERS intensity of 1L MoS_2_, paving the way for a tunable 2D material-based SERS platform. In this paper, we mostly focused on tuning the Raman intensity of 1L MoS_2_ using the proposed flexible nanotip array sample. However, the hybrid platform of the 1L MoS_2_/nanotip sample could also be extended to sense other molecules.

## 2. Materials and Methods

**Figure 1:**
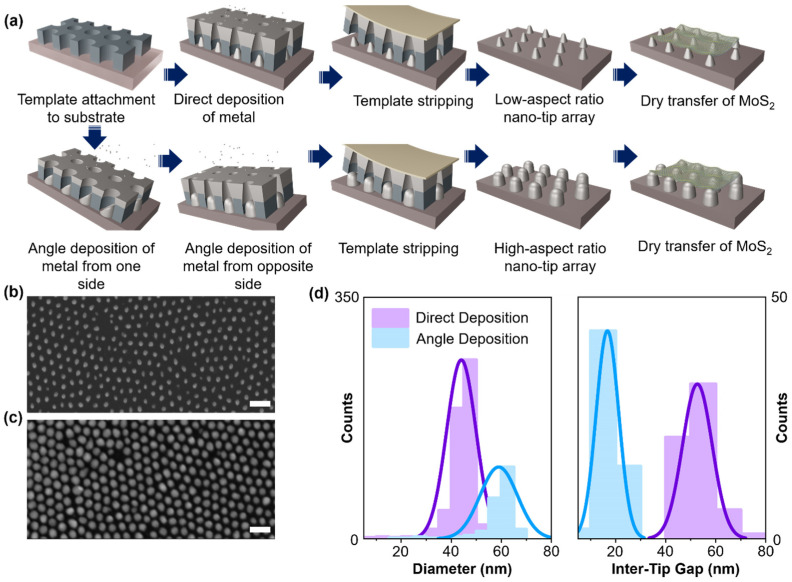
**Flexible nanotip array.** (**a**) Schematic diagram of fabrication process of flexible nanotip array. SEM images of (**b**) low and (**c**) high aspect ratio Ag nanotip array fabricated by direct and angle deposition, respectively (scale bar: 100 nm). (**d**) Distribution of diameter and inter-tip gap of nanotip array sample.

To realize the dynamic SERS platform, we fabricated a metallic nanotip array on polyethylene terephthalate (PET) substrate. The detailed fabrication is schematically described in Figure 1a. Briefly, at first, a large area (5 mm × 5 mm) of anodized aluminum oxide (AAO) template containing hole arrays of ~70 nm diameter with an interpole distance about ~100 nm is transferred to PET [28] (see Supplementary Information S1). Using the same AAO template, we fabricated two different types of samples: (1) low-aspect ratio nanotip and (2) high-aspect ratio nanotip. A direct deposition (normal to substrate) of Ag metal (~100 nm) leads to the low-aspect ratio nanotip array [29], whereas the low angle deposition (2 degree) of metal onto the AAO template leads to a high-aspect ratio nanotip array (Figure 1a and Supplementary Information S1). After metal deposition, we stripped the AAO template from the substrates using a Kapton tape to obtain the final sample containing the nanotip array. Figure 1b,c shows scanning electron microscopy (SEM) images of direct and angle-deposited Ag nanotips, respectively, which confirms the good coverage of the nanotip (see Supplementary Information S3 for cross-sectional images). Angle-deposited nanotips have higher coverage with ~70 nm diameter and shorter inter-tip distance of ~20 nm, as shown in Figure 1d. In the case of direct deposition, Ag nanoparticles cannot be deposited at the corner of the AAO template due to the shadow effect of nanoholes [30] (see Supplementary Information S2). However, in the angle deposition process, Ag nanoparticles are well deposited on the corner of the AAO template, resulting in Ag nanotips with high coverage. Finally, 1L MoS_2_ is transferred to the Ag nanotip/PET and to the bare PET substrate (Supplementary Information S4).

## 3. Results and Discussion

**Figure 2:**
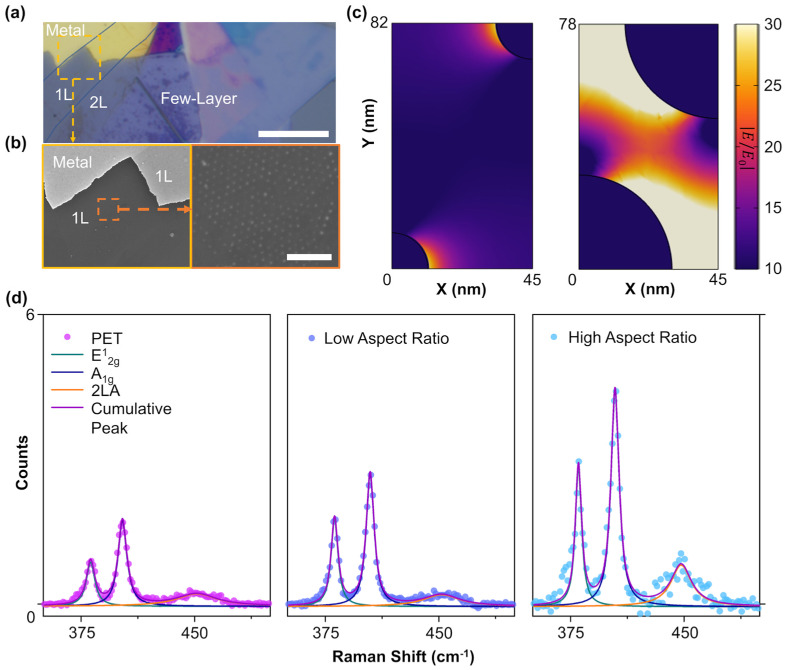
**Surface-enhanced Raman scattering of 1L MoS_2_ enabled by nanotip array.** (**a**) Optical microscope image of monolayer, bilayer (2L), and few-layer MoS_2_ on nanotip array. Scale bar: 10 um. (**b**) Top view FESEM images of nanotip covered with 1L MoS_2_. Scale bar: 500 nm. (**c**) FEM simulation of field confinement in between nanotips of low (left) and high (right) aspect ratio. (**d**) Raman spectra of 1L MoS_2_ on PET (left), low aspect ratio (middle), and high aspect ratio nanotips (right).

Figure 2a shows the optical microscope (OM) image of monolayer (1L), bilayer, and few-layer molybdenum disulfide (MoS_2_) flakes transferred onto the Ag nanotip with some parts overlapping the Ag metal film region. The FESEM image of the sample (area inside the yellow dashed line box) is shown in Figure 2b. The highly magnified FESEM image shown in Figure 2b confirms that 1L MoS_2_ is transferred well onto Ag nanotips. The height profile extracted from the atomic force microscope (AFM) image (Supplementary Information S5) further confirms the thickness of 1L MoS_2_ to be ~0.62 nm, which is in good agreement with the previously reported data [31,32,33]. At first, we conducted finite element method (FEM) electromagnetic simulation of low and high aspect ratio nanotip arrays, as shown in Figure 2c. For the low aspect ratio sample, the field is mostly confined to the tip region, which would affect the Raman intensity of 1L MoS_2_. For the high aspect ratio, since the tips are closer, the field around these tips interacts with each other. This eventually confines more light in between the tips, thereby enhancing the Raman signal even more as compared to the low aspect ratio nanotip sample. This enhancement is demonstrated experimentally as well. The Raman spectroscopy of the sample is measured using a commercial confocal Raman setup with a 532 nm excitation laser with 0.5 mW power to avoid any temperature-induced Raman peak shift [34] (see Supplementary Information S6). Figure 2d presents the Raman spectra of 1L MoS_2_ on the bare PET substrate, with the low and high aspect ratio Ag nanotip array samples. All the data were deconvoluted with three Lorentzian peaks to extract the peak position, full width at half maxima (FWHM), and intensity. The Raman spectra of 1L MoS_2_ on bare PET has two first-order vibrational peaks, i.e., the E^1^_2g_ peak at 384.27cm^−1^ due to the in-plane vibration of molybdenum (Mo) and sulfur (S) in the opposite direction, and the A_1g_ peak at 405.82 cm^−1^ due to the out-of-plane vibration of S [35,36]. The difference between these two peaks is ~21.55 cm^−1^, which is in accordance with the previous data for 1L MoS_2_ [37]. A third peak at 451.76 cm^−1^ is 2LA (longitudinal acoustic phonon) mode and is observed due to double-resonance Raman (DRR) scattering [38].

For SERS intensity, we checked the intensity of the Raman signal of the E^1^_2g_, A_1g_, and 2LA peak. The intensity for the 1L MoS_2_/high-aspect ratio nanotip is enhanced by a factor of ~2.7 times compared to bare 1L MoS_2_/PET (Supplementary Information S7). To further demonstrate our proposed tunable SERS platform, we used 1L MoS_2_ transferred on a high aspect ratio flexible nanotip array sample, since it offers enhanced intensity as compared to the low-aspect ratio nanotip array in its flat condition (Figure 2d).

**Figure 3:**
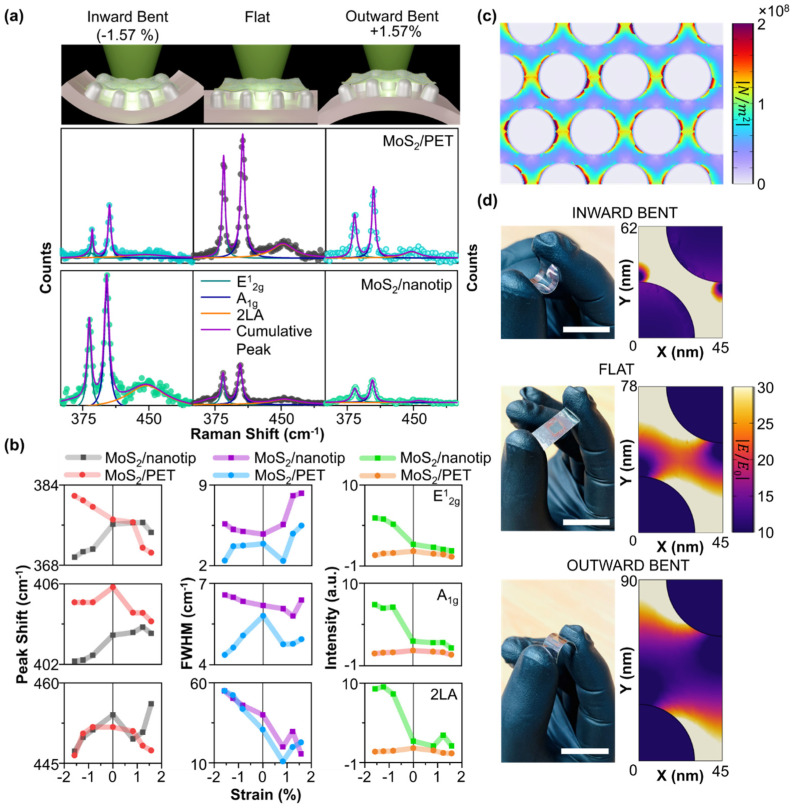
**Tunable SERS platform based on 1L MoS_2_/Nanotip.** (**a**) Schematic diagram describing tuning methodology of SERS of 1L MoS_2_/nanotip (top), strain-dependent Raman spectra of 1L MoS_2_/PET (middle), and 1L MoS_2_/nanotip (bottom). (**b**) Extracted strain-dependent Raman mode peak position, FWHM, intensity of 1L MoS_2_/PET, and 1L MoS_2_/nanotip. (**c**) FEM simulation of nanotip array showing stress concentration in between tips under inward bending. (**d**) Digital images of sample (left) and FEM simulation (right) of field confinement in between tips under inward bent (top), flat (middle), and outward bent (bottom) conditions (scale bar: 20 mm).

The schematic diagram of the method to tune the SERS intensity of the sample is presented in Figure 3a. As the sample is on a flexible substrate, we can apply both compressive (inward bent) and tensile (outward bent) strain to change the overall gap width in between nanotips, which ultimately increases or decreases the localized field intensity (Figure 3a). The strain-controlled field confinement eventually manipulates the SERS intensity of 1L MoS_2_. To avoid any artifacts due to changes in experimental condition, we fabricated all the samples at the same time with similar dimensions and measured them under similar bending conditions. Furthermore, the laser was focused on the same spot on 1L MoS_2_ for all the measurements at different bending conditions (see Supplementary Information S6). The detailed calculation of bending strain is presented in Supplementary Information S9. The sample was bent systematically with strain magnitudes of 0.82%, 1.22%, and 1.57% in both inward (negative sign) and outward (positive sign) directions. Figure 3b and Supplementary Information S10 present the strain-dependent Raman spectra of 1L MoS_2_/PET and 1L MoS_2_/nanotip. The extracted peak positions (mode peak), FWHM, and intensities are summarized in Figure 3c.

In the flat condition, for 1L MoS_2_/PET, the peak positions of E^1^_2g_, A_1g_, and 2LA were 384.27 cm^−1^, 405.82 cm^−1^, and 451.76 cm^−1^, respectively. For the 1L MoS_2_/nanotip, the respective peaks positions shifted to 384.04 cm^−1^, 403.44 cm^−1^, and 454.06 cm^−1^. While we observed a small shift (−0.06%) in the E^1^_2g_ peak position, the A_1g_ peak shifted by −0.58%. Since the E^1^_2g_ peak was insensitive to the substrate features, the small peak shift is due to the additional strain induced by the nanotip. The huge change in the A_1g_ peak is due to the charge doping from the nanotip to the 2D material [39,40]. The 2LA peak is red-shifted by 0.5%. At flat conditions, both FWHM and SERS intensity of the 1L MoS_2_/nanotip is increased because of the enhanced strain and field localization in between nanotips.

The mode peak E^1^_2g_ of 1L MoS_2_/PET is shifted by +0.3% and −0.4% when it is under compressive and tensile strain of 1.57%, respectively, as observed by other researchers [41,42]. When the 1L MoS_2_/nanotip is under compressive strain, E^1^_2g_ is red-shifted by 0.4%, which is the opposite of what we observed in 1L MoS_2_/PET. To explain this anomaly, we consider that under the flat condition, 1L MoS_2_ is fixed on top of the nanotip as a tent, without slippage. Under compression, the contact area between 1L MoS_2_ and the metal increases. As 1L MoS_2_ approaches the metal surface, it takes the shape of the nanotip, and tensile strain is only exerted on 1L MoS_2_ at the tip of the metal [25], which explains the anomalous redshift of the E^1^_2g_ peak under compressive strain. There is a very small change (−0.02%) in the E^1^_2g_ peak due to tensile strain on the 1L MoS_2_/nanotip. The out-of-plane vibration mode (A_1g_) is shifted by −0.18% and −0.42% for compressive and tensile strains of the same magnitude (1.57%) for 1L MoS_2_/PET, respectively. However, it is shifted by −0.31% (compressive strain) and 0.02% (tensile strain) for the 1L MoS_2_/nanotip. When the 1L MoS_2_/nanotip is bent inwardly, 1L MoS_2_ becomes much closer to the Ag nanotip, thereby causing n-type doping of 1L MoS_2_, as confirmed by the redshift of the A_1g_ peak. For the 2LA peak, the shift is about ~−1.0% in both bending directions in the 1L MoS_2_/PET sample. The redshift is due to a change in the lattice constant, which affects the band structure of the material [43,44]. The charge doping and strain also affect the line width of the spectra. The FWHM values of these modes are inversely proportional to the phonon lifetime. We observe that on PET and nanotip, the FWHM of 2LA mode increases (decreases) with compressive (tensile) strain, which is in good agreement with previous results [45]. Under compressive strain, A_1g_ and E^1^_2g_ peak broadening is observed due to electron–phonon scattering [46] and tensile strain [47], respectively, on the nanotip array. On the other hand, there is an increase in phonon lifetime in the 1L MoS_2_/PET substrate, which leads to a decrease in FWHM. However, for outward bending, the effect of uniaxial tensile strain is dominant rather than doping, leading to an increase in the FWHM of both the optical phonons in the 1L MoS_2_/nanotip [48,49].

**Figure 4:**
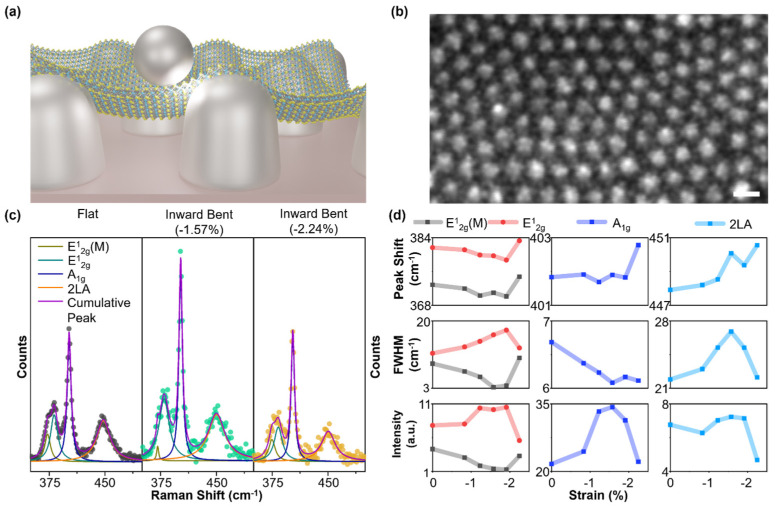
**Tunable SERS platform based on nanoparticles/1L MoS_2_/nanotip.** (**a**) Schematic diagram describing the tunable SERS platform based on nanoparticles/1L MoS_2_/nanotip. (**b**) SEM image of nanoparticle on nanotip sample (scale bar: 100 nm). (**c**) Compressive strain-induced Raman spectra of nanoparticles/1L MoS_2_/nanotip. (**d**) Extracted strain-dependent Raman mode peak position, FWHM, and intensity of nanoparticles/1L MoS_2_/nanotip.

In terms of SERS performance, the strain-controlled intensity change in Raman modes is also investigated. For 1L MoS_2_/PET, the intensities of all mode peaks decreased significantly by ~75% for both directions of bending. This decreasing tendency is not desirable for a highly sensitive tunable SERS platform. On the other hand, for 1L MoS_2_/nanotip, the intensities of the mode peaks E^1^_2g_, A_1g_, and 2LA are tuned from −45.6%, −40%, and −33.3% to +186.7%, +216.6%, and +374.3% by simply applying strain from +1.57 % (tensile) to −1.57% (compressive). Please note that it was surprising to observe that the tuning range was in increasing order from E^1^_2g_ to 2LA, which needs further study. We performed a finite element method (FEM) simulation of the structural and electromagnetic field of the nanotip array, as shown in Figure 3d. From the structural simulation, it was found that there is enormous strain localization in between the tips when bending strain is applied, which might cause the inter-tip gap width to increase or decrease. The electromagnetic field simulation confirms the increase or decrease in field confinement when the nanotips are closer or far away (Figure 3d). In pursuit of enhancing the SERS intensity, we deposited Ag nanoparticles using the e-beam evaporation method on the 1L MoS_2_/nanotip array to fabricate nanoparticle/1L MoS_2_/nanotip geometry [50]. These nanoparticles decorated on the 1L MoS_2_/nanotip would not only exert additional strain but also enhance the field confinement in the gap created by it. The schematic diagram of the sample is presented in Figure 4a. Shown in Figure 4b is the SEM image of the Ag nanoparticle deposited on the bare nanotip array on PET. From the image, it can be observed that the nanoparticles are decorated homogeneously on the surface of the tip. We also performed image processing of the FESEM image to isolate the nanoparticles and statistically determine the Ag nanoparticle size as ~13 nm (see Supplementary Information S11). The strain-dependent Raman spectra for the nanoparticle/1L MoS_2_/nanotip sample are shown in Figure 4c, Supplementary Information S12. We applied compressive strain to the sample to enhance the Raman signal. We observed the E^1^_2g_ mode to be split into two peaks at 372.85 cm^−1^ and 381.66 cm^−1^, suggesting additional strain due to nanoparticles in flat condition [20]. The extracted parameter corresponding to E^1^_2g_(M), E^1^_2g_, A_1g_, and 2LA mode is summarized in Figure 4d. Similar to 1L MoS_2_/nanotip, there is minimal change in the peak positions of E^1^_2g_ and A_1g_. However, E^1^_2g_(M) and 2LA peaks shifted by −0.73% and +0.6%, respectively. The FWHM of the E12g and 2LA peak increased, whereas it decreased for E^1^_2g_(M) and A_1g_. It is interesting to point out that the intensity of the SERS signal was greatly enhanced as compared to the 1L MoS_2_/nanotip with the inclusion of nanoparticles. The significant enhancement in the intensity of the Raman peaks is due to stronger field localization between the nanoparticle and the nanotip separated by 1L MoS_2_. Beyond −1.57% of strain, the intensity, peak position, and FWHM of all the modes reverted to their initial values. This might be due to the damage or sliding of 1L MoS_2_ by excessive strain [25].

**Figure 5:**
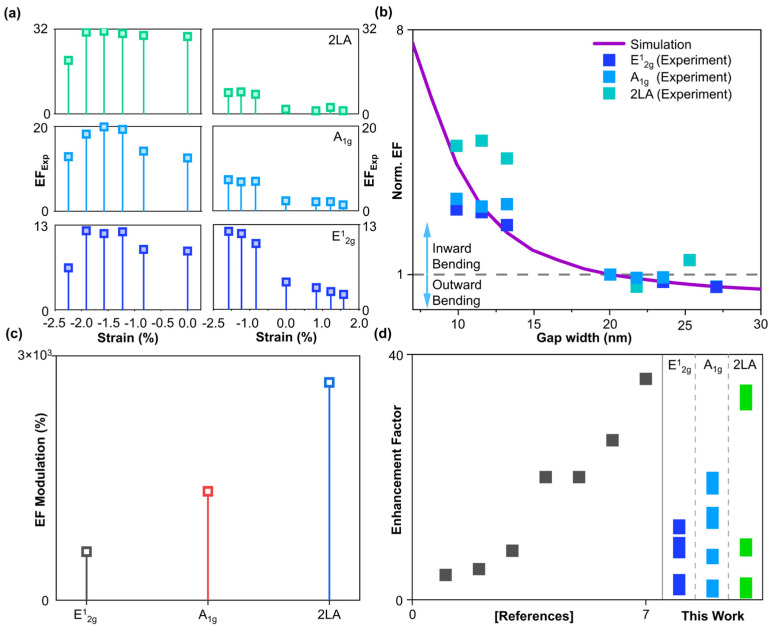
**Active control of enhancement factors.** (**a**) Strain dependent enhancement factors of nanoparticle/1L MoS_2_/nanotip and 1L MoS_2_/nanotip. (**b**) Depth of modulation of enhancement factor. (**c**) Finite element simulation of 1L MoS_2_/nanotip for various inter-dot gap sizes. (**d**) Literature survey of enhancement factor of 1L MoS_2_ and comparison with this work.

Enhancement factor is an important parameter for validating the SERS performance (Supplementary Information S13) [51]. The experimental enhancement factor (EF_Exp_) is calculated by (Supplementary Information S11)

(1) $$EF_{Exp}=\frac{I_{SERS}}{I_{Reference}}$$

where $I_{SERS}$ is the surface-enhanced Raman intensity (in our case, nanoparticles/1L MoS_2_/nanotip and 1L MoS_2_/nanotip) and $I_{Reference}$ is the Raman intensity of the bare 1L MoS_2_ on the PET substrate. The highest enhancement factors are calculated to be 12, 20, and 34.1 for E^1^_2g_, A_1g_, and 2LA modes, respectively, at −1.57% (inward) strain for nanoparticles/1L MoS_2_/nanotip. We assume that the significant enhancement in 2LA Raman mode is due to exciton–plasmon coupling in 1L MoS_2_ [49]. To find out the strain-dependent inter-tip gap width change, the simulated enhancement factor for the different gap width is normalized and compared with normalized EF_exp_, as shown in Figure 5b (Supplementary Information S14 and S15). From the data, it can be concluded that there is an overall gap width increase (decrease) of 10 nm for inward (outward) bending. The modulation of depth of the enhancement factor is of the order 10^3^ as compared to the lowest enhancement factor of 1L MoS_2_/nanotip at outward bending (Figure 5c). We could also achieve initial intensity at flat after the bending experiment (Supplementary Information S16), showing the reconfigurability of the platform. We checked the intensity at various spots in the nanoparticle/1L MoS_2_/nanotip sample (Supplementary Information S17). We observed similar SERS signals from five different spots, suggesting a large-area SERS platform. A comparative study between our work and the reported work is presented in Supplementary Information S18, and the respective graph is shown in Figure 5d. Although some of the work reported a good enhancement factor, our work shows the ability to control the enhancement factor from low to high for first- and second-order Raman signals.

## 4. Conclusions

In summary, we demonstrated a mechanically active SERS platform based on nanoparticles/1L MoS_2_/nanotip and 1L MoS_2_/nanotip fabricated on a PET substrate. We recognized three different modes, E^1^_2g_, A_1g_, and 2LA, in the Raman spectra of 1L MoS_2_ on bare substrates and nanotip arrays. The peak position and FWHM were affected by the external strain induced. More importantly, the SERS intensity was enhanced (reduced) significantly with inward (outward) bending of the 1L MoS_2_/nanotip sample. To enhance the signal even more, silver nanoparticles were deposited on the 1L MoS_2_/nanotip sample, forming nanoparticles/1L MoS_2_/nanotip geometry. With the external inward bending, this sample offered an enhancement factor as high as 35. The overall enhancement factor was tuned from 1.23 to 35, showing an excellent tunability of the SERS platform. The change in intensity (or enhancement factor) is due to the change in gap width, which is confirmed by FEM structural and electromagnetic simulation. Our work may find applications in developing future tunable devices for energy, sensing, and opto-electronics.

## Data Availability

The original contributions presented in the study are included in the article/Supplementary Material, further inquiries can be directed to the corresponding authors.

## Supplementary Materials

The following supporting information can be downloaded at: https://www.mdpi.com/article/10.3390/bios14120619/s1, Supplementary Information S1. Transfer of anodized aluminum oxide (AAO) template to PET substrate; Supplementary Information S2. Fabrication of low and high-aspect ratio Ag nanotip array; Supplementary Information S3. SEM images of flexible nano-tip array; Supplementary Information S4. Dry transfer of MoS_2_ to Ag nanotip array; Supplementary Information S5. Characterization of MoS_2_ after transfer on PET substrate; Supplementary Information S6. Confocal Raman setup and measurement details; Supplementary Information S7. Extracted parameters from Raman spectra of MoS_2_/PET and MoS_2_/nanotip array; Supplementary Information S8. Experimental enhancement factor of MoS_2_/PET and MoS_2_/nanotip array; Supplementary Information S9. Calculation of bending strain of nanotip array on PET substrate; Supplementary Information S10. Detail strain dependent Raman data for MoS_2_/PET and MoS_2_/nanotip; Supplementary Information S11. FESEM images of nanoparticle/MoS_2_/nanotip; Supplementary Information S12. Detail strain dependent Raman data of nanoparticle/1L MoS_2_/nanotip; Supplementary Information S13. Enhancement factor calculation from experiment and simulation; Supplementary Information S14. FEM simulation of electromagnetic wave of low-aspect ratio nanotip array; Supplementary Information S15. FEM simulation of electromagnetic wave of high-aspect ratio nanotip array; Supplementary Information S16. Reproducibility of Raman spectra of 1L MoS_2_/nanotip after bending experiment; Supplementary Information S17. Raman spectra of nanoparticle/1L MoS_2_/nanotip measured on various spots; Supplementary Information S18. Plasmon resonance for nanotip and nanoparticle/nanotip array; Supplementary Information S19. Comparison of reported enhancement factor of MoS_2_ with current work [52,53,54,55,56,57,58,59,60].
