# Supplementary material for: Active Surface-Enhanced Raman Scattering Platform Based on a 2D Material–Flexible Nanotip Array
Source: Biosensors (Basel). 2024 Dec 15;14(12):619. doi: 10.3390/bios14120619 (PMC11674311; doi:10.3390/bios14120619)
Supplement: Supplementary file 1 [file biosensors-14-00619-s001.zip › biosensors-3335861-Supplementary Materials.pdf]

Supplementary Information

Active Surface-Enhanced Raman Scattering Platform Based on a 2D Material–Flexible Nanotip Array

**Yong Bin Kim [1,†], Satyabrat Behera [2,†], Dukhyung Lee [2], Seon Namgung [2], Kyoung Duck Park [1], Dai Sik Kim [2,*]
and Bamadev Das [2,*]**

[1] Department of Physics, Pohang University of Science and Technology (POSTECH), Pohang 37673, Republic of Korea; life0944@postech.ac.kr (Y.B.K.); parklab@postech.ac.kr (K.-D.P.)

[2] Department of Physics and Quantum Photonics Institute, Ulsan National Institute of Science and Technology (UNIST), Ulsan 44919, Republic of Korea; satyabrat2020@unist.ac.kr (S.B.); dukhyung.lee@um6p.ma (D.L.); seon@unist.ac.kr (S.N.)

* Correspondence: bamadevdas0@gmail.com (B.D.); daisikikim@unist.ac.kr (D.S.K.)

† These authors contributed equally to this work.

SI1. Transfer of anodized aluminum oxide (AAO) template to PET substrate.

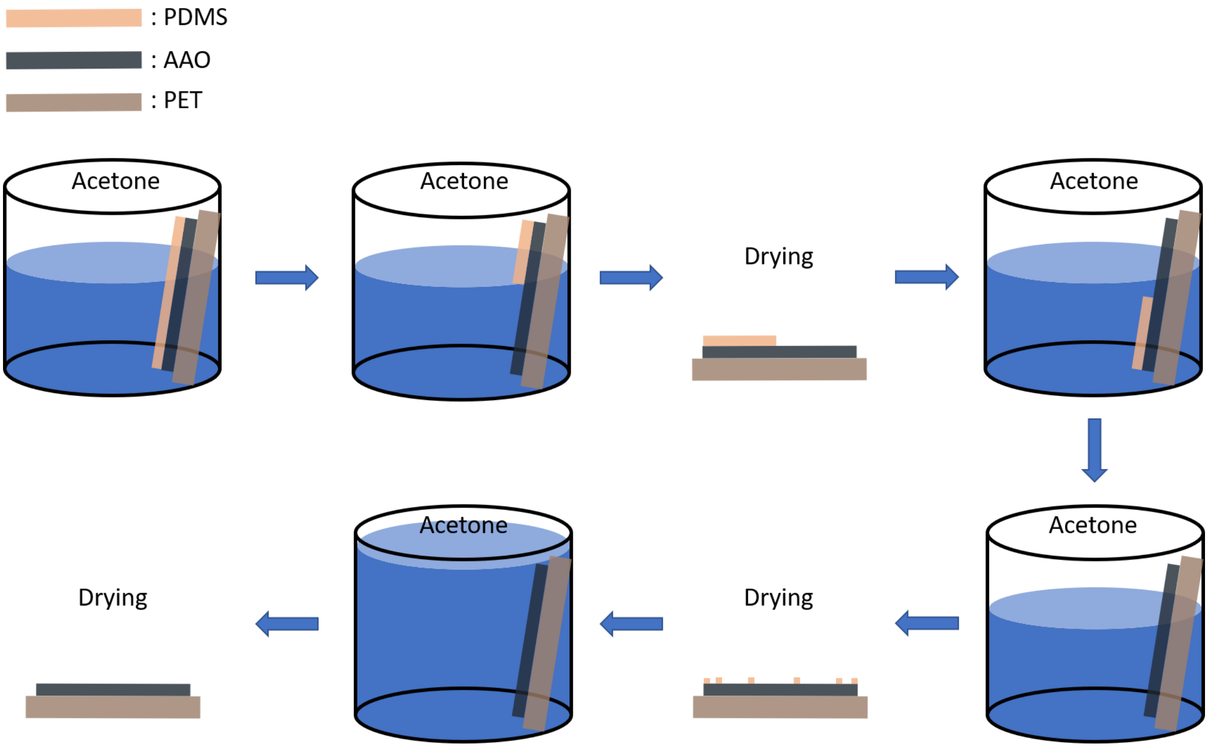

Figure S1. Schematic of fabricating AAO/PET substrate.

PET substrate was cut into 20 x 10 mm size. The AAO template (200 nm height, ~ 70 nm diameter hole array) was transferred onto PET substrate by PDMS assisted transfer meth od. PDMS was removed by dipping in acetone. Firstly, 60% of PMMA/AAO/PET substrate was dipped in acetone for 15 minutes, followed by dried in air. Secondly, remained part o f substrate was dipped in acetone for 15 minutes, then dried in air completely. Finally, to remove residual PMMA on substrate, whole part of AAO/PET substrate was dipped in ace tone for 15 minutes.

SI2. Fabrication of low and high-aspect ratio Ag nanotip array.

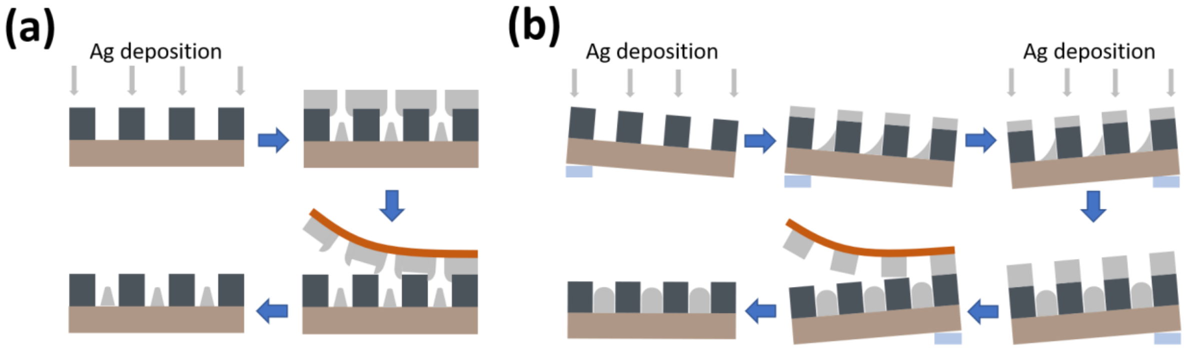

Figure S2. Schematic of direct (a) and angle deposition (b) process.

Ag nanotips were deposited on to AAO/PET substrate by electron beam evaporator. We f abricated two kind of samples, low and high aspect ratio Ag nano-tips using slow vapor deposition method which is speed of 0.4 Å/sec. For low-aspect ratio nanotip array, as sho wn in figure S3.(a), Ag particles were deposited normal to the surface of AAO/PET substra te. In this case, some particles are sticked on the side of AAO template. As a result, Ag p articles cannot be deposited at the corner of AAO template, which is called shadow effec t. For high-aspect ratio, as shown in figure S3.(b), AAO/PET substrate was leaned on the s lide glass which has 2 mm height to apply 2 degrees angle on substrate, which can avoid shadow effect. Due to deposition of Ag particles at the corner of AAO, Ag tips show hig her coverage than direct deposition method. Finally, AAO was removed using typical lift-off method.

SI3. SEM images of flexible nano-tip array.

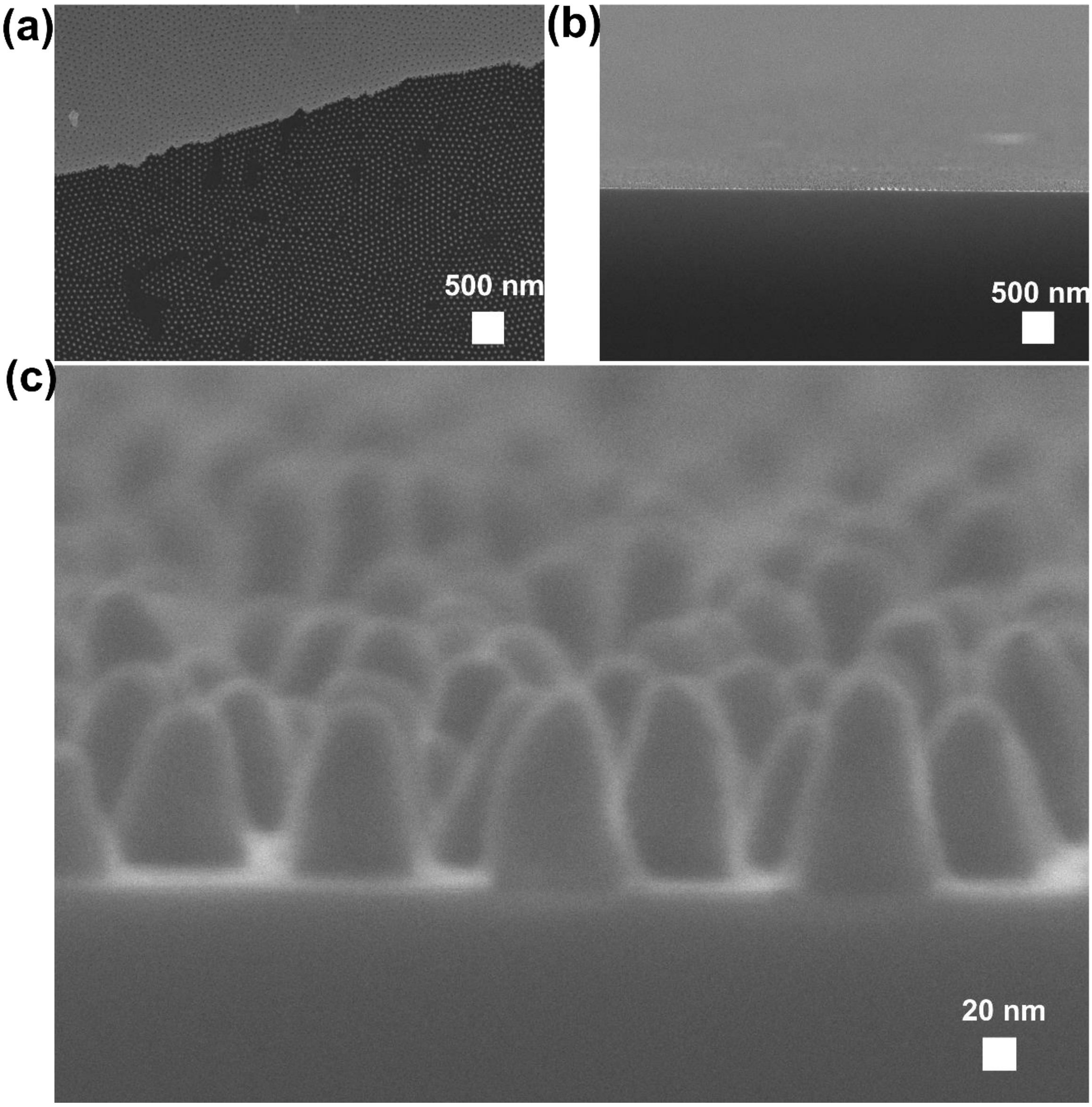

Figure S3. (a) Top view SEM image of low aspect ratio nanotip array and AAO template on Si substrate. (b) Low and (c) high magnified cross-sectional SEM image of nanotip arra y.

SI4. Dry transfer of MoS_2 to Ag nanotip array.

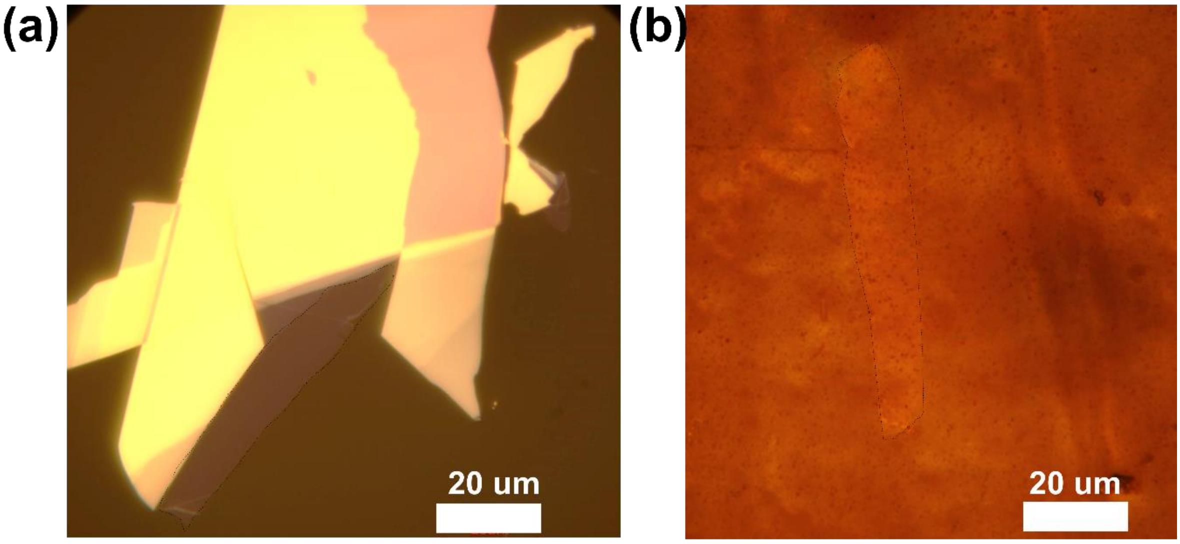

Figure S4. Optical microscope image of 1L-MoS_2 (a) after exfoliation on PDMS (b) after d ry transfer on nanotip array fabricated on PET substrate.

Initially MoS_2 flake is exfoliated onto a PDMS stamp by mechanical exfoliation wit h blue tape. The monolayer flake is identified by its contrast in the optical micros cope image. The PDMS stamp is attached to a glass slide connected to a micro m anipulator. By using a microscope the flake on the PDMS stamp is aligned and slo wly brought in contact with the Ag nanotip/PET substrate fixed on a heating stag e. The stage is heated to 80 degree Celsius for 1 hour to improve the 1L MoS_2 an d Ag nanotip adhesion. After an hour, the stage is cooled to room temperature a nd PDMS stamp is raised slowly in a way that the 1L MoS_2 stick onto the Ag nano tip/PET substrate.

SI5. Characterization of MoS_2 after transfer on PET substrate.

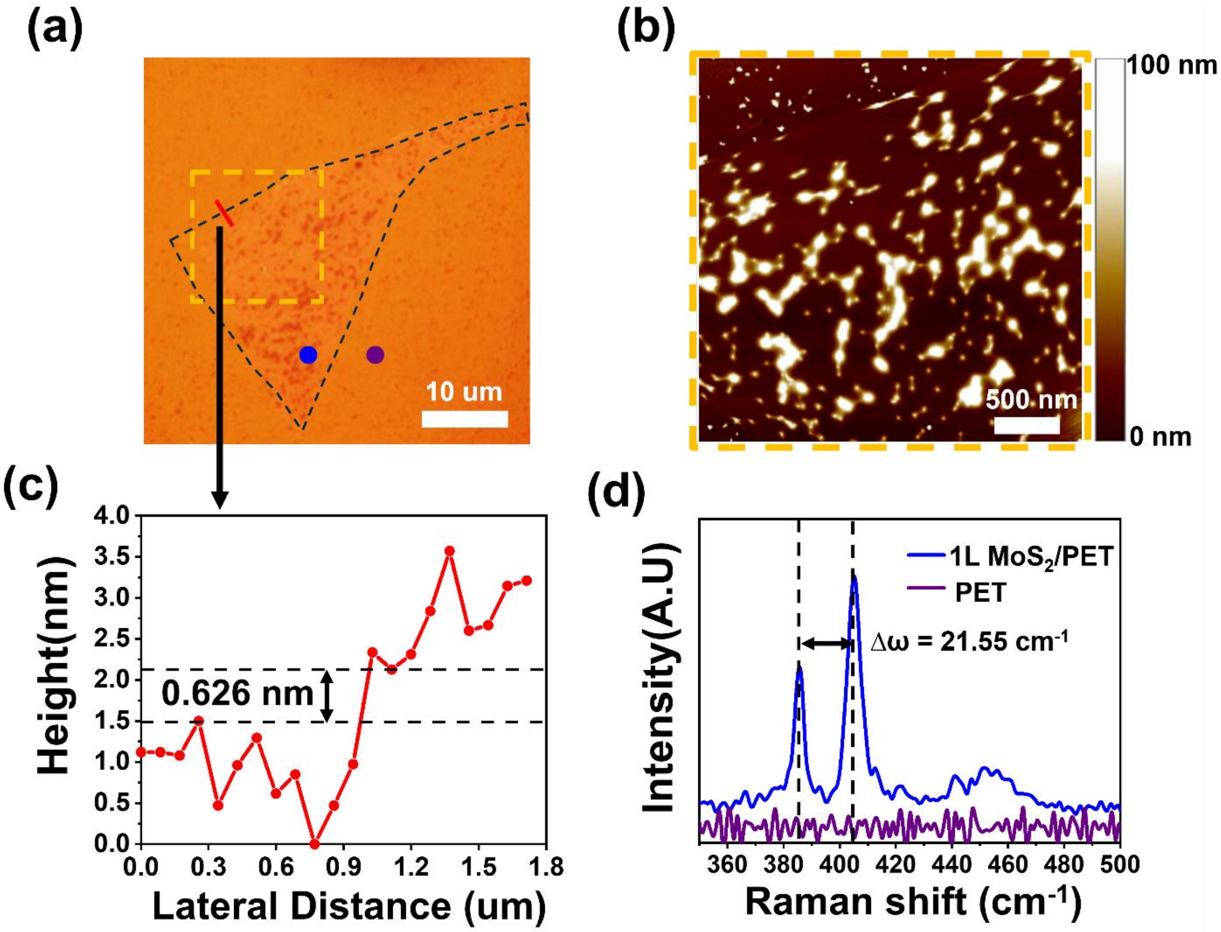

Figure S5. Optical microscope image of MoS_2 flake after transfer on PET substrate. The monolayer region is outlined in both the figures. (b) AFM image of the region enclosed in yellow box in figure (a). (c) AFM height profile along the red line in figure (a). (d) Raman spectrum from the MoS_2 region(blue dot) and PET region(purple dot) in figure (b).

The thickness of our MoS_2 flake after dry transfer on PET substrate was characterized using both AFM measurement and Raman spectroscopy. Figure S5 (a) shows the optical microscope image of MoS_2 flake after transfer on PET substrate. As shown in figure S5 (b) we performed AFM measurement on the region highlighted(yellow square) in (a). Note that the PDMS residue on MoS_2 can be observed from the AFM data. Figure S5 (c) shows the height profile along the red line marked in (a). Raman spectrum measured from the MoS_2 region(blue dot) and PET substrate(purple dot) is shown in figure S5 (d).

SI6. Confocal Raman setup and measurement details.

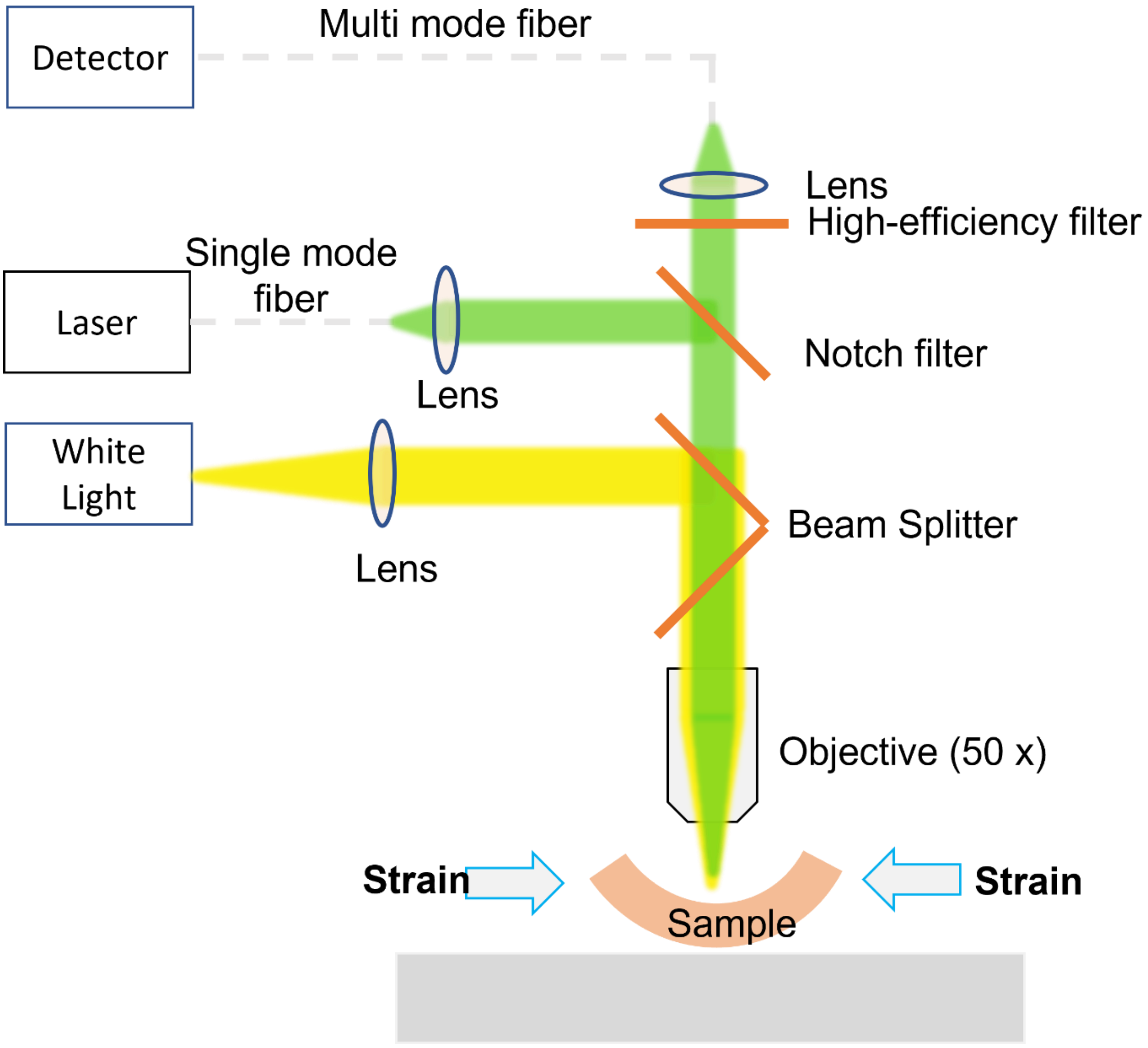

Figure S6. Schematic diagram of confocal setup.

We measured strain dependent Raman spectra using a commercial confocal Raman setup (WITEC), shown in Figure S5. A custom made holder is used for applying strain while me asuring Raman spectra of the sample. The laser (532 nm) is focused on the same spot on the sample for various bending strain conditions.

SI7. Extracted parameters from Raman spectra of MoS_2 /PET and MoS_2/nanoti p array.

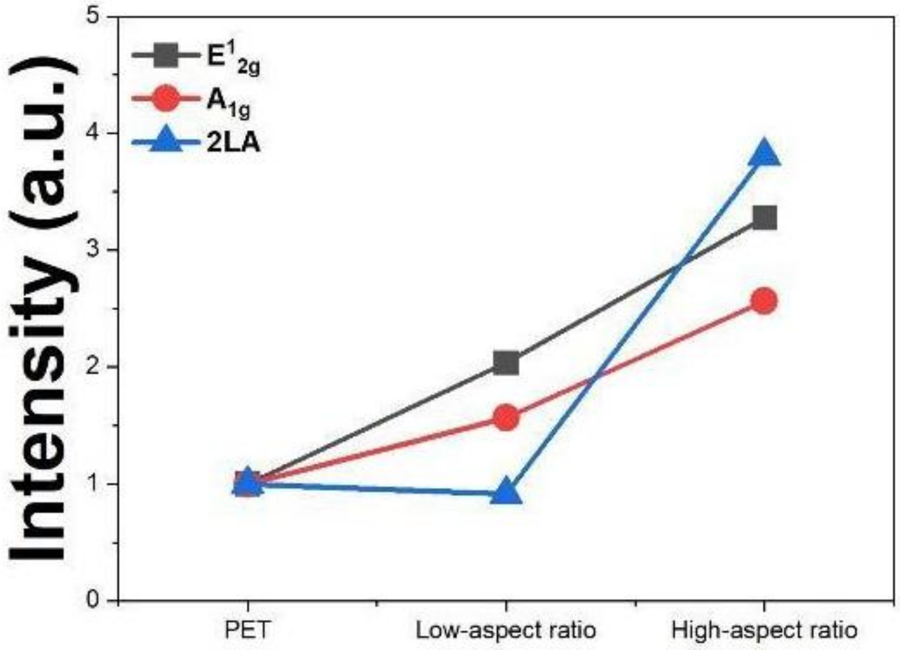

Figure S7. Extracted intensity of 2LA, E^1_{2g}, A_{1g} mode for 1L MoS_2 on PET, low and high as pect ratio nanotip.

SI8. Experimental enhancement factor of MoS_2 /PET and MoS_2 /nanotip array.

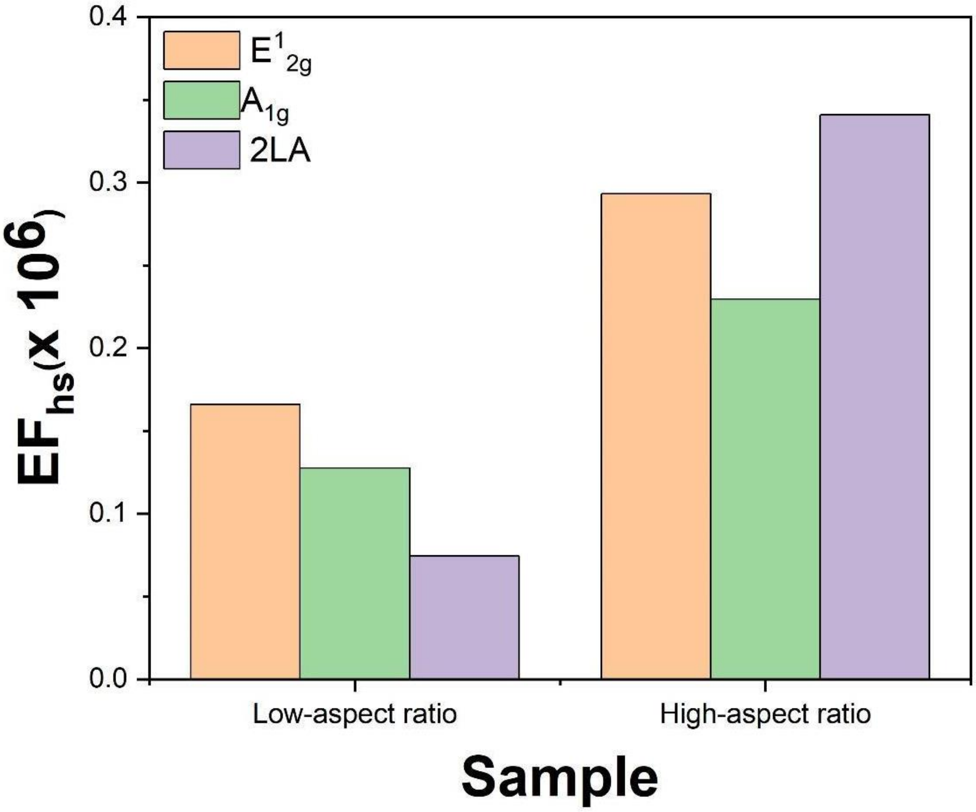

Figure S8. Experimental enhancement factor of E^1_{2g}, A_{1g}, 2LA mode of MoS_2 on low and high-aspect ratio nanotip array.

SI9. Calculation of bending strain of nanotip array on PET substrate.

The bending radius of curvature is calculated by following the analytical expression (S1), which is related to the actual length (L_0) of the sample and the length (L_x) of the sample after being bent by moving the stage movement (x).

The bending radius of curvature of the sample can be calculated using the general expression

$$R_c = \frac{(1+\ y'^2)^{3/2}}{y''} \qquad Eq.S1$$

Assuming the shape of the bent substrate to be sinusoidal $y = kx$ having two unknowns A and k. We apply two constraints to find these constants. First one is $y(x = 0) = y(x = L_x) = 0$ and the second constraint is $L_x = \int_0^{L_x} \sqrt{(1 + (\frac{dy}{dx})^2)} dx$. Now solving these two constraints finding two unknowns and putting the sinusoidal equation in equation S1, the radius of curvature at the center of the sample is derived as

$$R_c = {L_x}^2/(\pi^2 A) \qquad Eq.S2$$

Finally, the strain can be calculated by

$$\varepsilon\ (\%) = \frac{h}{2R_c} \times\ 100 \qquad Eq.S3$$

Where h is the thickness of PET substrate (250 um).

SI10. Detail strain dependent Raman data for MoS_2/PET and MoS_2/nanotip.

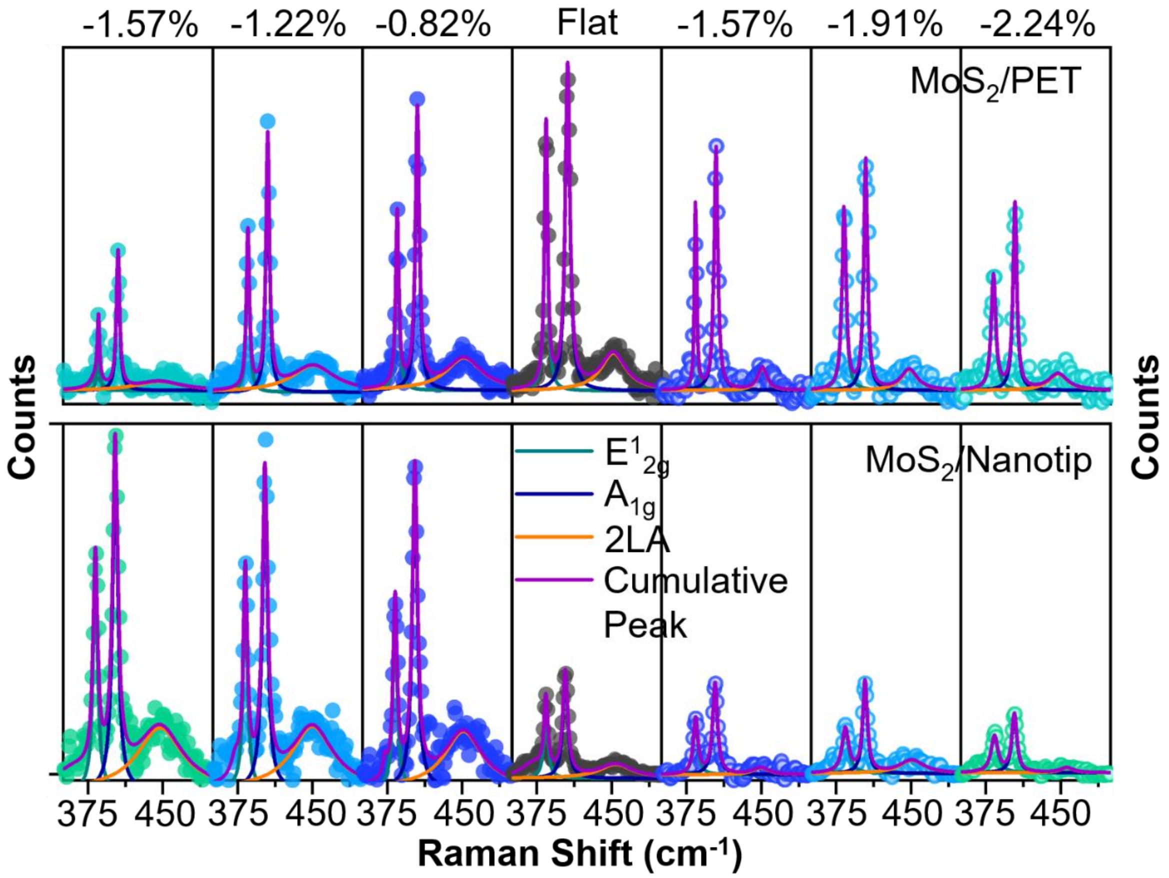

Figure S10. Strain dependent Raman spectra of 1L MoS_2/PET (top) and 1L MoS_2/nanotip (bottom).

SI11. FESEM images of nanoparticle/MoS_2/nanotip

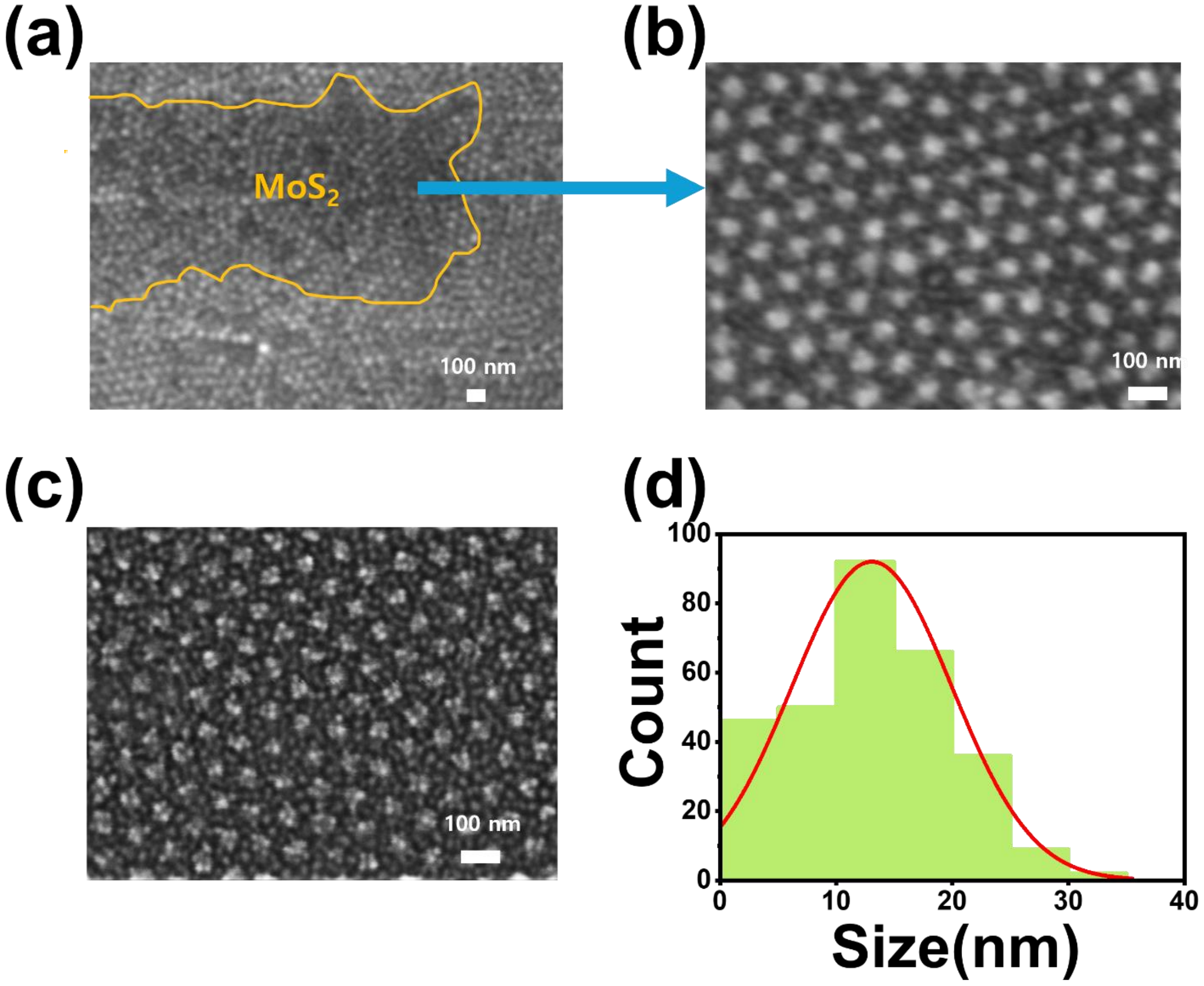

Figure S11. (a)Low (left) and (b)high (right) magnified FESEM image of nanoparticle/1L MoS_2/nanotip array. (c) Image processing performed on (b) for filtering the nanoparticle features and analyze its diameter. (d) Size distribution statistics of nanoparticles. From the distribution we can conclude that Ag nanoparticles of diameter ~13 nm are deposited on 1L MoS2/nanotip array.

SI12. Detail strain dependent Raman data of nanoparticle/1L MoS_2/nanotip.

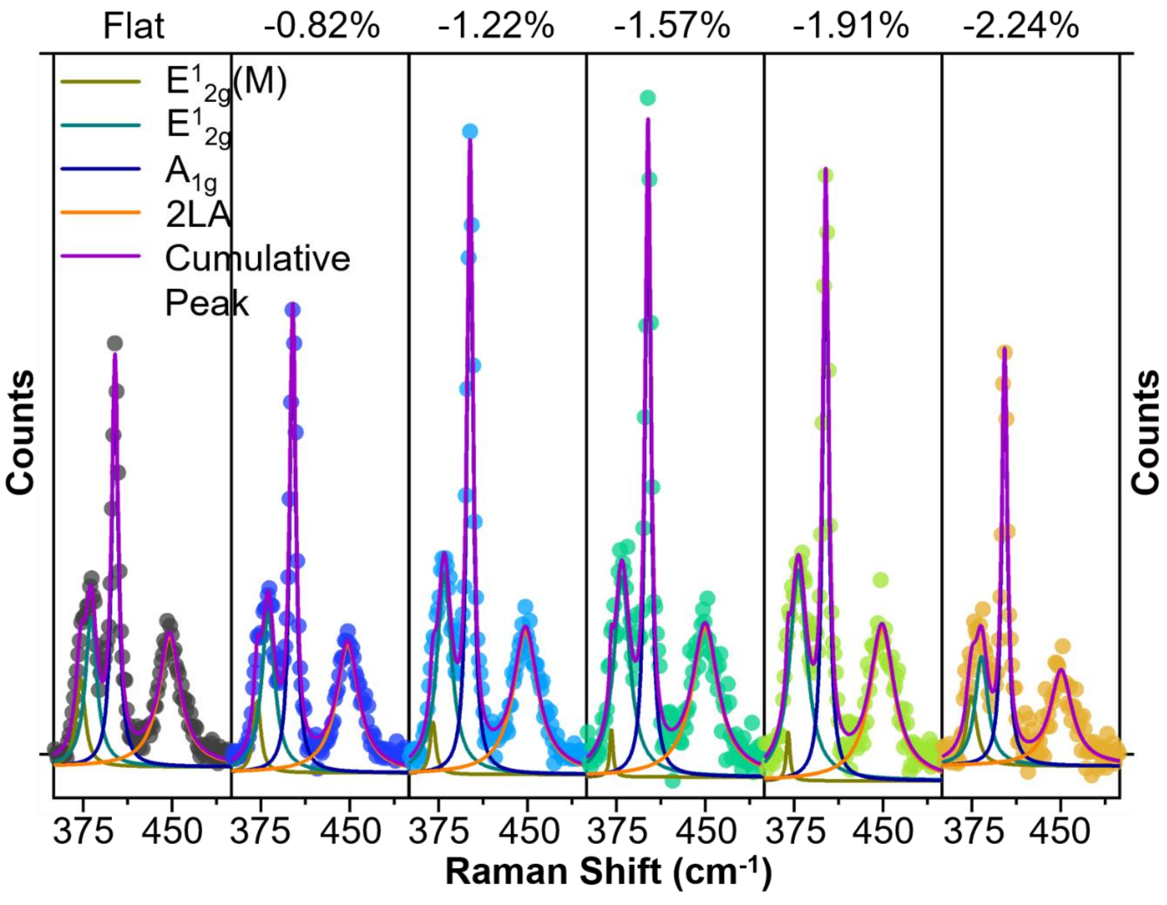

Figure S12. Compressive strain induced Raman spectra of nanoparticles/1L MoS_2/nanotip.

SI13. Enhancement factor calculation from experiment and simulation.

The experimental enhancement factor (EF_{Exp}) can be calculated by

$$EF_{Exp} = \frac{I_{SERS}}{I_{Ref}} \qquad Eq.S4$$

Where I_{SERS} is the measured Raman intensity of MoS_2 on nanotip array and I_{Ref} is the measured Raman intensity of MoS_2 on PET substrate. However, to compare t he experimental enhancement factor with simulated data, we normalized the expe rimental enhancement factor with hot-spot area[7][9][10], which is given below

$$EF_{hotspot} = \frac{I_{SERS}}{I_{Ref}} \times \frac{A_{hotspot}}{A_{Laser}} \qquad Eq.S5$$

$A_{hotspot}$ and A_{Laser} is the area of hotspot and laser spot size. To calculate $EF_{hotspot}$, we used laser spot diameter of 20 um and hotspot area calculated from SEM im ages of nanotip array.

SI14. FEM simulation of electromagnetic wave of low-aspect ratio nanotip arr ay.

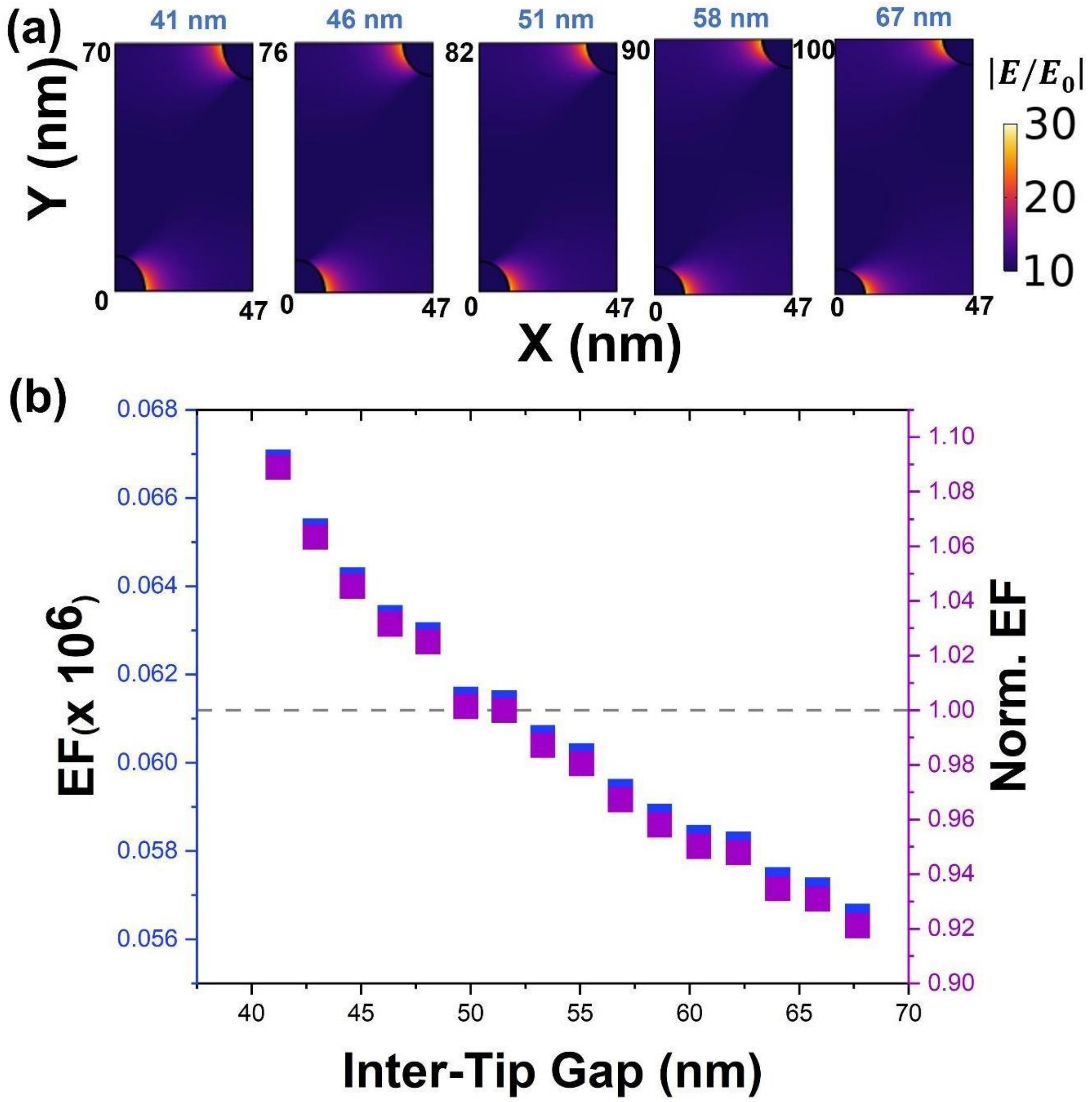

Figure S13. (a) FEM simulation of electromagnetic field confinement in low-aspect ratio n anotip array of various inter tip-gap width. (b) Simulated enhancement factor of low-aspe ct ratio nanotip of various inter-tip gap width.

SI15. FEM simulation of electromagnetic wave of high-aspect ratio nanotip arr ay.

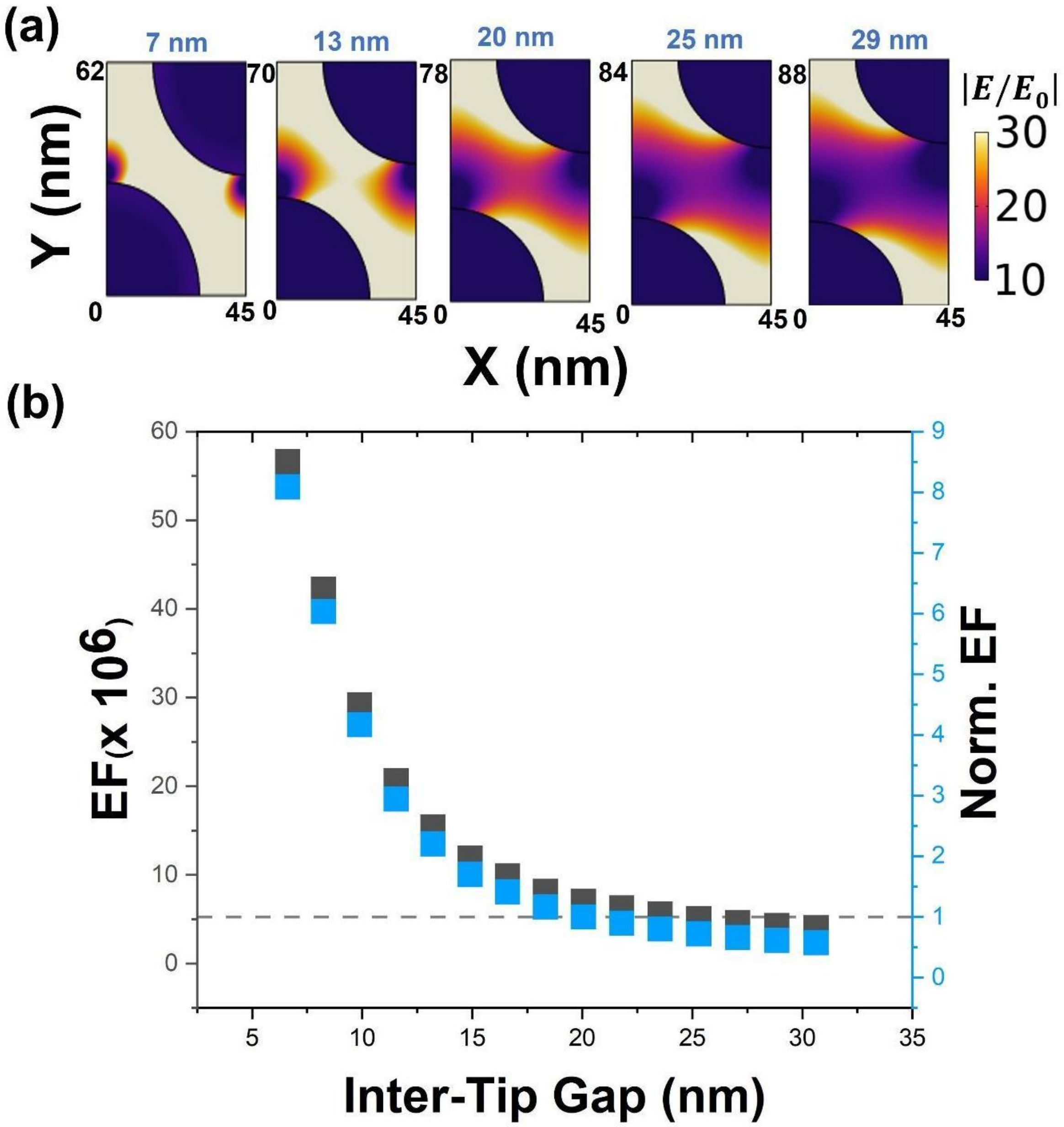

Figure S14. (a) FEM simulation of electromagnetic field confinement in high-aspect ratio nanotip array of various inter tip-gap width. (b) Simulated enhancement factor of high-as pect ratio nanotip of various inter-tip gap width.

SI16. Reproducibility of Raman spectra of 1L MoS_2/nanotip after bending exp eriment.

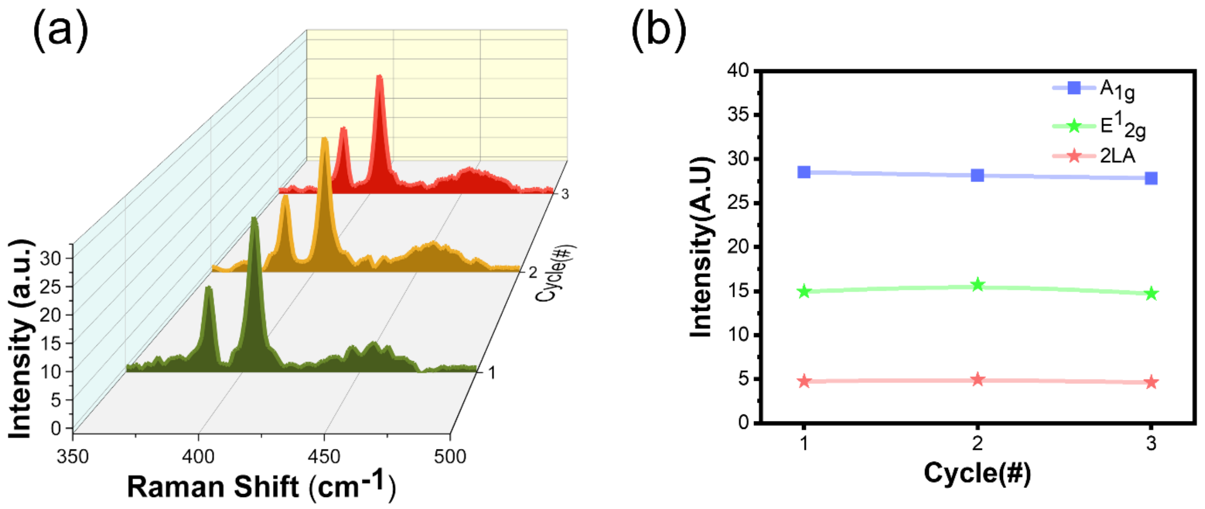

Figure S15. (a) Measured Raman spectra and (b) Intensities of A_{1g}, E^1_{2g} and 2LA Raman m odes of MoS_2/high aspect ratio nanotip array after each bending cycle.

SI17. Raman spectra of nanoparticle/1L MoS_2/nanotip measured on various sp ots.

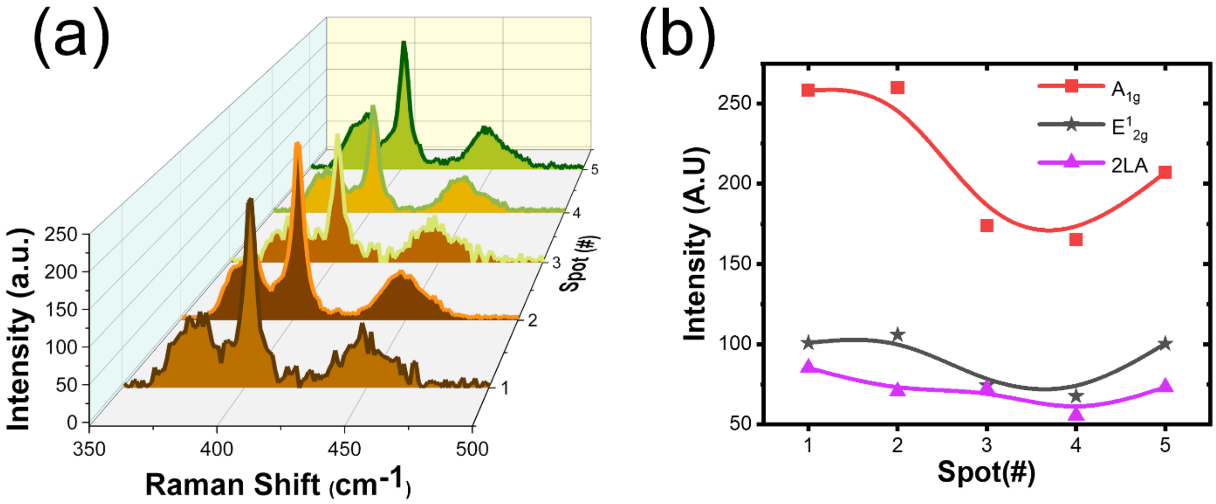

Figure S16. (a) Measured Raman spectra and (b) Intensities of A_{1g}, E^1_{2g} and 2LA Raman m odes of nanoparticle/1L MoS_2/high aspect ratio nanotip array on different spots.

SI18. Plasmon resonance for nanotip and nanoparticle/nanotip array.

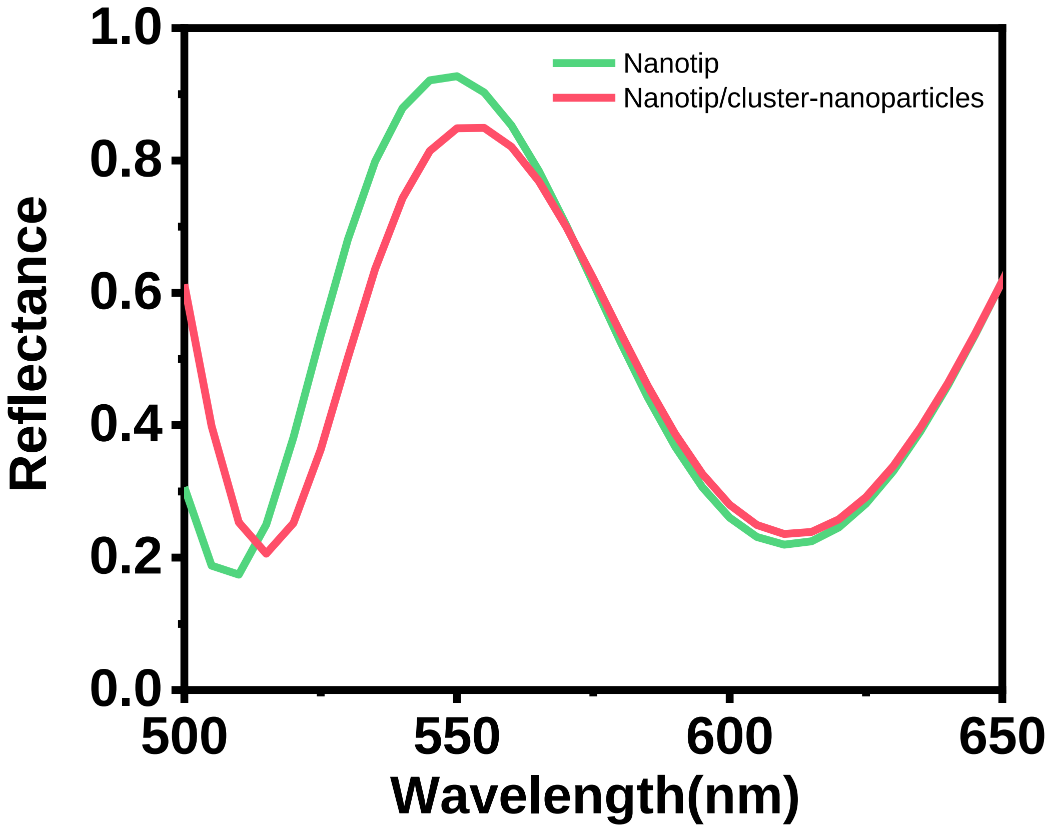

Figure S18. Simulated reflectance spectrum of nanotip and nanoparticle/nanotip array.

SI19. Comparison of reported enhancement factor of MoS_2 with current work.

References	Sample	Enhancement Factor			Type of Platform
		E^1_{2g}	A_{1g}	2LA	
1	Few layer MoS_2 decorated with Au nanorod	3	3.8	--	Passive
2	Bulk and Few layer/Ag Grating	4.85	4.05	--	Passive
3	1L MoS_2/on Ag nanoprisms and nanoparticles	8.6	5	--	Passive
4	1L MoS_2/topological insulator nanoparticle	--	8	--	Passive
5	1L MoS_2/Ag nanorod array	8	20	--	Passive
6	MoS_2 flakes decorated with Au nanoparticles	20	20	--	Passive
7	1L MoS_2/Ag nanogrooves	22	26	--	Passive
8	1L MoS_2/nanodimer	--	36	--	Passive
This work	MoS_2/nanotip nanoparticle/MoS_2/nanotip	1.74-12.1	1.38-18.16	1.23-34	Tunable

Table S1. Comparative study showing the reported enhancement factor of MoS_2 with cur rent work.

References

[1] I. Irfan, S. Golovynskyi, O. A. Yeshchenko, M. Bosi, T. Zhou, B. Xue, B. Li, J. Qu, and L. Seravalli, *Plasmonic Enhancement of Exciton and Trion Photoluminescence in 2D MoS2 Decorated with Au Nanorods: Impact of Nonspherical Shape*, Phys. E Low-Dimensional Syst. Nanostructures **140**, 115213 (2022).

[2] H. Zheng, M. Li, B. Chen, B. Sangho, C. M. Joseph, K. Gangopadhyay, and S. Gangopadhyay, *Surface-Plasmon-Enhanced Raman and Photoluminescence of Few-Layers and Bulk MoS2 on Silver Grating*, in *Conference on Lasers and Electro-Optics* (Optica Publishing Group, San Jose, California, 2016), p. JW2A.111.

[3] D. Li, H. Lu, Y. Li, S. Shi, Z. Yue, and J. Zhao, *Plasmon-Enhanced Photoluminescence from MoS2 Monolayer with Topological Insulator Nanoparticle*, **11**, 995 (2022).

[4] L. Su, L. Bradley, Y. Yu, Y. Yu, L. Cao, Y. Zhao, and Y. Zhang, *Surface-Enhanced Raman Scattering of Monolayer Transition Metal Dichalcogenides on Ag Nanorod Arrays*, Opt. Lett. **44**, 5493 (2019).

[5] P. Farhat, M. O. Avilés, S. Legge, Z. Wang, T.-K. Sham, and F. Lagugné-Labarthet, *Tip-Enhanced Raman Spectroscopy and Tip-Enhanced Photoluminescence of MoS2 Flakes Decorated with Gold Nanoparticles*, J. Phys. Chem. C **126**, 7086 (2022).

[6] J. H. Kim, J. Lee, S. Park, C. Seo, S. J. Yun, G. H. Han, J. Kim, Y. H. Lee, and H. S. Lee, *Locally Enhanced Light–Matter Interaction of MoS2 Monolayers at Density-Controllable Nanogrooves of Template-Stripped Ag Films*, Curr. Appl. Phys. **33**, 59 (2022).

[7] Q. Hao, J. Pang, Y. Zhang, J. Wang, L. Ma, and O. G. Schmidt, *Boosting the Photoluminescence of Monolayer MoS2 on High-Density Nanodimer Arrays with Sub-10 Nm Gap*, Adv. Opt. Mater. **6**, 1700984 (2018).

[8] Z. Wang, Z. Dong, Y. Gu, Y.-H. Chang, L. Zhang, L.-J. Li, W. Zhao, G. Eda, W. Zhang, G. Grinblat, S. A. Maier, J. K. W. Yang, C.-W. Qiu, and A. T. S. Wee, *Giant Photol*

uminescence Enhancement in Tungsten-Diselenide–Gold Plasmonic Hybrid Structures, Nat. Commun. **7**, 11283 (2016).

[9] G. M. Akselrod, T. Ming, C. Argyropoulos, T. B. Hoang, Y. Lin, X. Ling, D. R. Smith, J. Kong, and M. H. Mikkelsen, *Leveraging Nanocavity Harmonics for Control of Optical Processes in 2D Semiconductors*, Nano Lett. **15**, 3578 (2015).
